# Supplementary material for: CMOS-compatible ising machines built using bistable latches coupled through ferroelectric transistor arrays
Source: Sci Rep. 2023 Jan 27;13:1515. doi: 10.1038/s41598-023-28217-8 (PMC9883258; doi:10.1038/s41598-023-28217-8)
Supplement: Supplementary file 1 — Supplementary Information. [file 41598_2023_28217_MOESM1_ESM.pdf]

Supplementary Information

CMOS-Compatible Ising Machines built using Bistable Latches Coupled through Ferroelectric Transistor Arrays

Antik Mallick[1], Zijian Zhao[2], Mohammad Khairul Bashar[1], Shamiul Alam[3], Md Mazharul Islam[3], Yi Xiao[4], Yixin Xu[4], Ahmedullah Aziz[3], Vijaykrishnan Narayanan[4], Kai Ni[2], Nikhil Shukla[1]*

[1]Department of Electrical and Computer Engineering, University of Virginia, Charlottesville, VA 22904, USA

[2]Department of Electrical and Microelectronic Engineering, Rochester Institute of Technology, Rochester, NY 14623, USA.

[3] Department of Electrical Engineering and Computer Science, University of Tennessee, Knoxville, TN 37996, USA

[4]Department of Computer Science & Engineering, Pennsylvania State University, State College, PA 16801, USA

*email: ns6pf@virginia.edu

Supplementary Figures

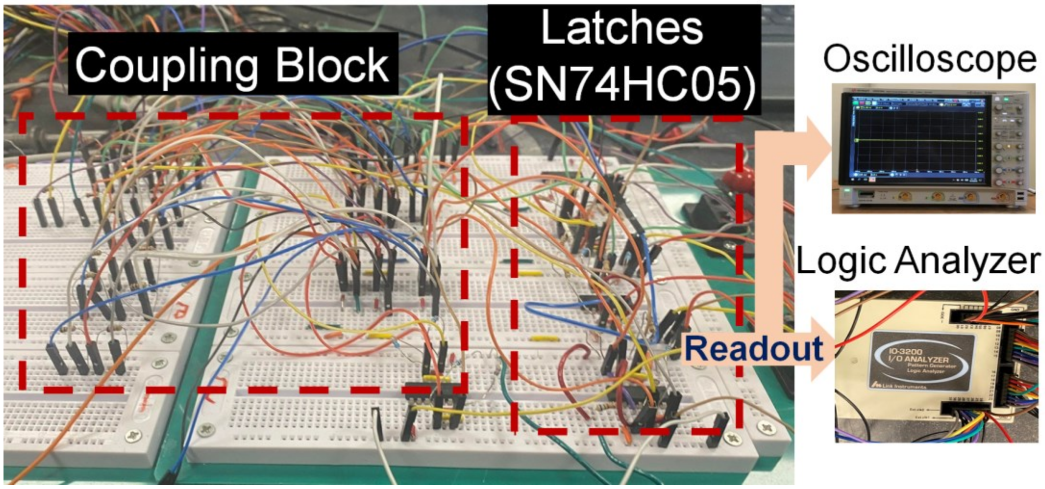

Supplementary Figure 1| Picture of the experimental setup. The latches are implemented using IC74HC05. The outputs are measured using an oscilloscope and logic analyzer.

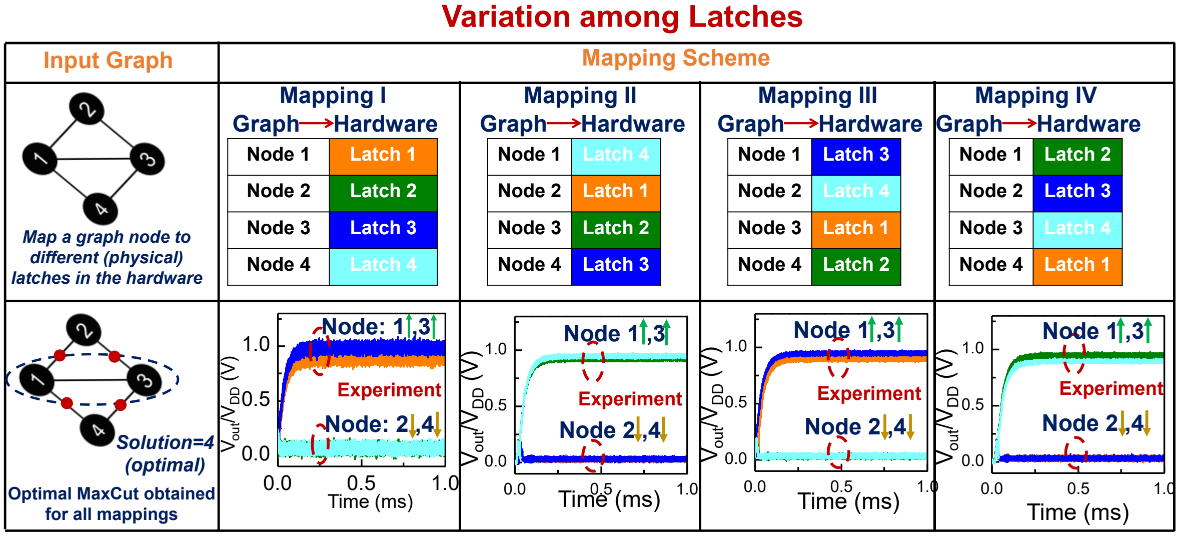

Supplementary Figure 2| Evaluation of the impact of asymmetry among the CMOS Latches. Observed MaxCut solution when the nodes in the input graph are mapped to different physical latches in the hardware. Optimal solution is observed for all the mappings indicating that the observed behavior is not the result of inherent asymmetry among the latches.

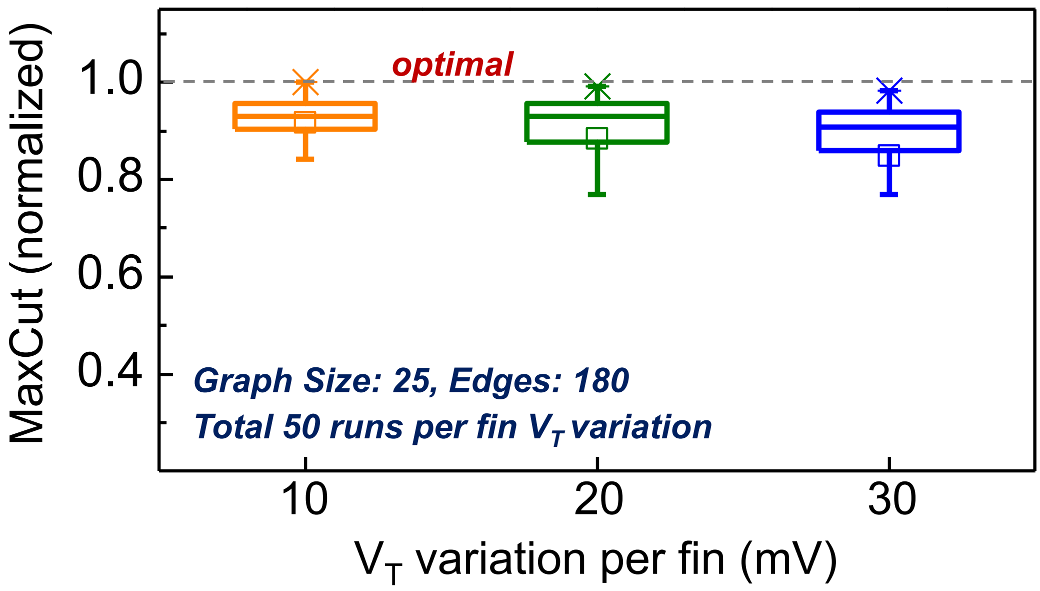

Supplementary Figure 3| Effect of Threshold Voltage Variation. Simulation showing the effect of threshold voltage variation (per fin) in the latches on the MaxCut solution computed by the latch based Ising machine array; Monte Carlo simulations (3σ, 50 runs) are performed for each case. Box plot results; center: median; box: interquartile range (IQR); whiskers: 1.5× IQR)

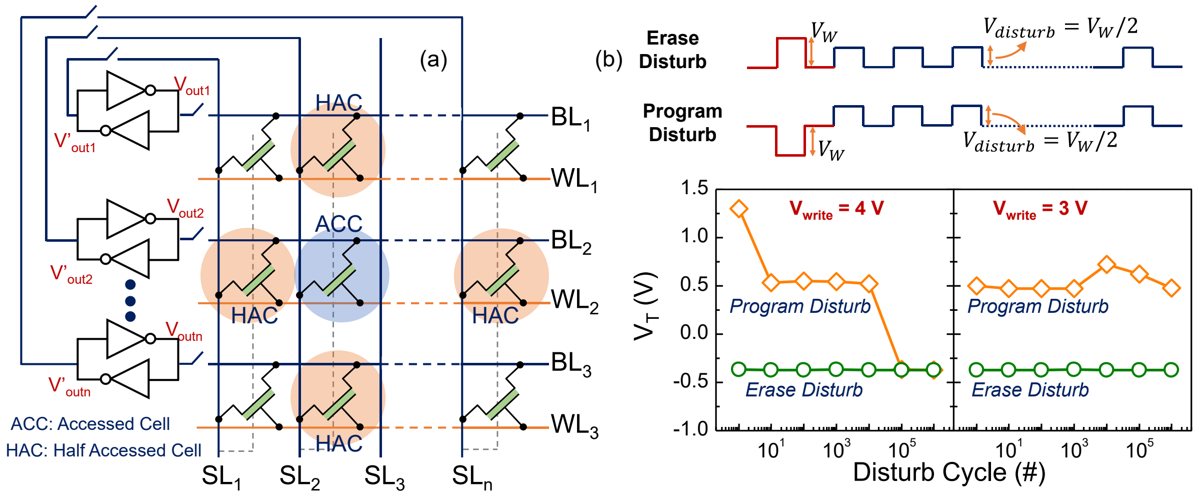

Supplementary Figure 4| Write Disturb in FeFET Array. (a) Schematic of the 1T-FeFET used in the Ising machine implementation. The (blue) circled cells indicate an accessed cell while the (orange) circled cells indicate the half-accessed cells. (b) Evolution of the threshold voltage in the HAC cells corresponding to repetitive program and erase of the ACC cell for two different write voltages, V_W= 4V and 3V.

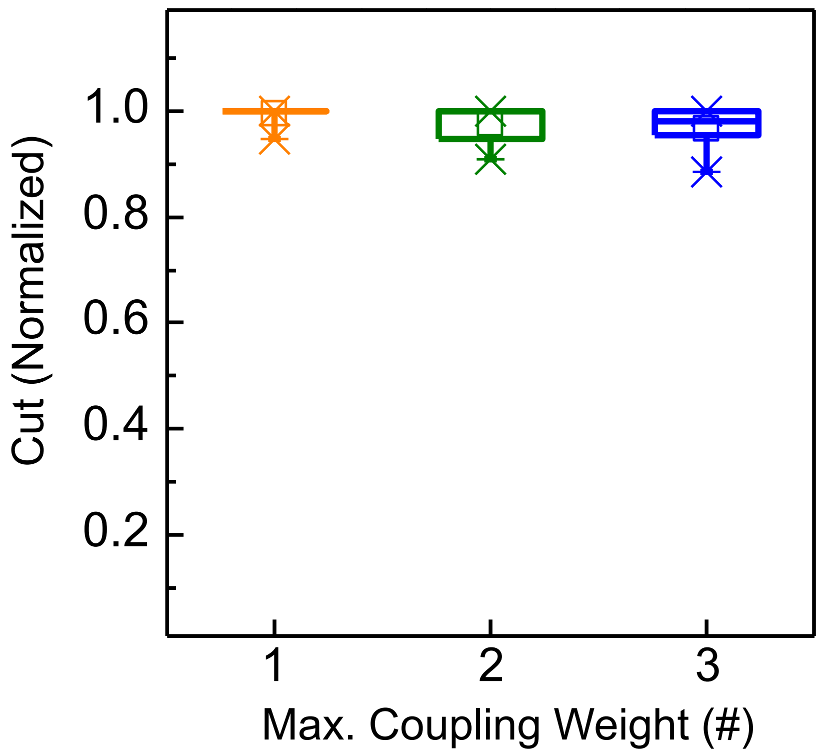

Supplementary Figure 5| Solving Weighted Graph Instances. Cuts computed by the system (simulation) for weighted graphs with up to 2-bit weights. The graphs are randomly generated and categorized according the maximum weight in the graph. 30 graphs (size 10 nodes) were tested in total.

Supplementary Notes

Supplementary Note 1. Experimental Setup

Supplementary figure 1 shows the experimental setup for measuring the dynamics of resistively coupled latches as an Ising machine. The latch-based Ising spin is implemented using discrete CMOS inverters (IC SN74HC05N), which are subsequently, coupled through external resistors that behave as coupling elements. The outputs are measured using a digital oscilloscope (Keithley DSOS104A) and a logic analyzer (Link Instruments IO-3200). The inverters are biased using a supply voltage of 5V sourced through a Keithley 2400 source meter.

Supplementary Note 2. Evaluation of Asymmetry among Latches

To rule out the possibility that the observed output states of the latches are caused by inherent asymmetry (arising from variation) amongst them, we map the nodes in the illustrative graph to different physical latches, as shown in supplementary figure 2. For instance, in mapping I, nodes 1, 2, 3, 4 are mapped to latches 1, 2, 3, 4, respectively, and in mapping II, nodes 1, 2, 3, 4 are mapped to latches 4, 1, 2, 3, respectively. It can be observed that the latches settle to optimal MaxCut solution irrespective of the mapping, indicating that the latch asymmetry does not dominate the outputs of the latches in the experiment.

Supplementary Note 3. Effect of Threshold Voltage Variation in FeFET coupled Latches

We explore the effect of threshold voltage (V_T) variation (expressed threshold variation per fin) in the transistors (used for the latches) on the behavior of the proposed Ising machine array (Fig. 5 of main text). The latches are constructed using 10nm PTM models (5-fin FinFET design is used), and the analysis is performed using a Monte Carlo approach (50 runs). It is observed from supplementary figure 3 that the system is able to compute high-quality MaxCut solutions in the presence of variation although the mean accuracy of the solution reduces with increasing V_T variation. Further, the simulations indicate that V_T variation >20 mV variation per fin can have a significant impact on the solution quality.

Supplementary Note 4. Write Disturb in FeFET Array

We program the FeFET array as a standard memory array by adopting the half-select ($V_W/2$) write scheme wherein a column-wise write operation is performed with a write voltage (V_W) of 3V (Fig. 5 of main text). The reason behind this choice of write voltage was to ensure that the half-accessed cells experience minimum write disturb i.e., unintentional change in threshold voltage is response to programming of other cells. It can be observed from supplementary figure 4b that the half-accessed cells show minimal disturb when V_W= 3V even after 10^4 cycles; in contrast, a write voltage of 4V induces significant disturb which would have an adverse impact on the programming operation.

Supplementary Note 5. Solving weighted graph instances

Supplementary figure 5 shows 30 graph instances with up to 2-bit weights (0,1,2,3; 4-states). The programming voltage used to achieve the desired conductance state (corresponding to the weight) was given by: $V_{prog} = K + \alpha.W_{ij}$, where $K = 3$, $\alpha = 0.2$ and W_{ij} represents the weight between node i and j. We observe that the mean accuracy of the cut (over all the 30 graph instances) is >97% of the optimal value.
